# PROTOCOL: Single‐track year‐round education for improving academic achievement in us k‐12 schools: results of a meta‐analysis

**DOI:** 10.1002/CL2.194

**Published:** 2018-10-24

**Authors:** Dan Fitzpatrick, Jason Burns

## Background

### The problem, condition or issue

Summer learning loss is a prominent concern in academic and public discussions of education. Summer learning loss refers to the fact that students forget material and show measurably decreased competency at the beginning of one school year than at the end of the preceding year. Concerns focus on not only what students forget over summer vacation, but also on the time that must be spent reviewing previously‐taught material at the beginning of each school year. Overall, summer learning loss is worse in math than in reading (Cooper, Nye, Charlton, Lindsay, & Greathouse, 1996), likely because students read but do not do math during the summer. Cooper et al.'s (1996) meta‐analytic estimate was that achievement declines by about one month of learning (.16 standard deviations in math and .11 in reading) during summer.

Summer learning loss appears to be worse for disadvantaged students. Research has documented that low‐income students lose ground to higher‐SES students during summer months when they cannot access school resources (Burkam, Ready, Lee, & LoGerfo 2004; Entwisle, Alexander, & Olson, 2001). The magnitude of this loss relative to their more‐advantaged peers is substantial: low‐income students lose as much as three months of learning in reading over the summer (Von Drehle, 2010). Summer learning loss among low‐income students may account for as much as two‐thirds of the income‐based achievement gap (Alexander, Entwisle, & Olson, 2007).

The documented losses for disadvantaged students are consistent with research on differences in summer resources and opportunities. Low‐income students typically attend lower‐performing schools than their wealthier counterparts, but the resource differential in summer may be even greater (Downey, von Hippel, & Broh, 2004). During summer, less affluent children watch more television, converse less with parents, and benefit from less daily parental involvement than wealthier students (Gershenson, 2013). Wealthier students, in contrast, are more likely to engage in more stimulating activities, like taking lessons, visiting libraries, and attending museums, than less affluent students (Alexander et al., 2007).

### The intervention

Year‐round education is seen as a way to combat summer learning loss by shortening or eliminating the long summer vacation. Year‐round education refers to the policy intervention of shortening summer break (and increasing the frequency and/or length of shorter breaks during the school year) in order to distribute instructional time more evenly throughout the year, while retaining the standard 180 instructional days. The National Association for Year‐Round Education (NAYRE) defines YRE by saying that it provides “more continuous learning by breaking up the long summer vacation into shorter, more frequent vacations throughout the year…The year‐round calendar is organized into instructional periods and vacation weeks that are more evenly balanced across 12 months than the traditional school calendar” (NAYRE, n.d.). One common calendar example alternates 45 instructional days (9 weeks) with 10 days (2 weeks) of vacation; this allocation of time is called a 45–10 calendar, and results in a summer vacation of around 6 weeks instead of 10 or more.

Year‐round education is sometimes conflated with other calendar and instructional reforms, so it is worth delineating how it is distinct from seemingly similar policies. YRE is distinct from a reform that is typically called extended year, which consists of adding days to the standard American school year of 180 days. YRE also does not refer to after‐school programming, tutoring, summer school for remediation, other summer programming, or lengthening the number of instructional hours in each school day. It refers exclusively to re‐allocating the 180 instructional days more evenly throughout the year.

Single‐track YRE calendars can differ from each other on two important axes. Single‐track YRE can be implemented in a variety of calendar structures—whether a calendar has 30 days of instruction followed by 5 of vacation (called 30–5), 45 days of instruction followed by 10 of vacation (45–10), 45–15, 60–20, or another alternative—which could moderate the impact of the calendar type on student achievement. Single‐track YRE calendars can also differ in the number of weeks to which summer vacation is shortened. Schools shorten their summer from the traditional 10 weeks to lengths ranging from 5 to 8 weeks; given the concern about summer learning loss, it would not be surprising for those lengths to moderate the effectiveness of single‐track YRE.

### How the intervention might work

The logic of year‐round education is fairly simple: if there are fewer weeks for students to forget material, then they will forget less during the summer, need to spend fewer weeks on review, and make more progress in the following year. The thinking of advocates is that the more‐frequent short breaks (e.g. of two weeks, in a 45–10 calendar structure) are not long enough to engender learning loss in the same way that lengthy summer vacations do. If correct, distributing vacations and schooling more evenly throughout the year would allow for students' year‐over‐year academic progress to increase, with no additional days of teaching.

### Why it is important to do the review

Two prior meta‐analyses have examined year‐round education's effect on academic achievement, primarily with subjects merged into a single outcome. Kneese (1996) included both studies with comparison groups and pre/post studies, and found a positive effect on achievement varying from +0.11 to +0.2 standard deviations depending on the exact model and analysis used. Kneese also stated that single‐track calendars appeared to have a larger effect than multi‐track calendars. Cooper, Valentine, Charlton, and Melson (2003) included only studies with comparison groups, and found an overall effect size of +0.06, but +0.11 for studies that used statistical or matching controls. Cooper et al. (2003) disaggregated by calendar type, and in their fixed‐effects unadjusted analyses found that, although multi‐track YRE had an effect size of just −0.01 (±.05), single‐track YRE had an effect size of +0.16.

These prior reviews provided important information on how YRE overall relates to student learning. However, the Cooper et al. (2003) study included research through 2000. Since 2001, in the NCLB and post‐NCLB era, schooling in America has experienced a broad array of shifts and interventions. These may have introduced systemic differences in the effect of YRE. Perhaps more importantly, the prior reviews focused on YRE overall, and only examined single‐track YRE as a whole compared to multi‐track YRE as a whole. By focusing only on single‐track year‐round education, I will be able not just to arrive at an overall effect size estimate, but also to begin observing both qualities that make single‐track YRE more effective and student populations for whom it is more effective.

The findings from this meta‐analysis may be able to guide decisions about whether single‐track YRE is an intervention that increases student achievement, and for schools with what type of students it is most likely to be effective.

## Objectives

Guided by prior research, this meta‐analysis examines single‐track YRE only. The main objective is to identify, across studies published in the post‐NCLB era, how single‐track YRE effects student learning. The first research question is therefore: (1) what is the estimated effect of single‐track YRE for math achievement and for reading achievement? The summer learning loss literature shows that disadvantaged students fall further behind their advantaged peers over the summer. This disparity points to the possibility that the effect size of YRE, which derives in part from mitigating summer learning loss, will differ for subgroups of students. Thus, the second research question is: (2) what is the effect size (of math and reading achievement) for only low‐income students and for only minority students? There may well also be differences in the effect of single‐track YRE based on the characteristics of the calendar as implemented. The third research question is therefore: (3) what is the relationship between characteristics of YRE (calendar structure, duration of the longest remaining break) and the effect size estimate?

## Methodology

### Criteria for including and excluding studies

#### Types of study designs

As is commonly the case in education research, I do not expect to encounter any experimental studies. Much research in this area is simply mean achievement comparisons at schools with similar demographic characteristics. In order to avoid excessively restricting the size of my final sample, I intend to include studies that use any approach to comparing academic achievement at traditional calendar schools versus single‐track year‐round schools. This is likely to include single‐track year‐round schools compared to a comparison group based on: matched school‐level characteristics, matched student‐level characteristics and geographic proximity (e.g. within a small county). I will exclude any studies that do *not* have achievement data. Many analyses are only of different average achievement (at one school or at multiple schools; sometimes using student‐level data and sometimes using school‐level data), so I will include these mean comparison data. I will also include multivariate observational and econometric studies with statistical controls, which for this meta‐analysis will typically mean OLS regression.

In general, I will apply an exclusion criteria that studies must include a comparison group; and this will *exclude* comparisons made to prior performance at the same school(s). Pre/post comparisons are not accepted in Campbell reviews. A subset of YRE evaluations use pre/post *cohort* designs (e.g. comparing the performance of students in Cohort 1, who were on a traditional calendar, to students in Cohort 2, who were on a year‐round calendar that was newly implemented, where Cohorts 1 and 2 are all enrolled students (in a given grade) at the *same* school). Because of that tension, I will conduct a sensitivity analysis of how including cohort‐comparison studies shifts the estimated average effect size.

#### Types of participants

Studies must be of K‐12 schooling (students) in the United States. The restriction to the United States will avoid introducing the confounding variable of number of school days. The restriction to K‐12 schooling will allow for effect estimates to be for commonly‐grouped primary and secondary education, without including studies examining modified school calendars in early childhood education, pre‐school, or college. Additionally, I intend to consider studies of whole schools or of only regular‐education students (who are in some cases the only students for whom achievement data are available), but not any studies of special education students.

#### Types of interventions

Year‐round calendars are not all the same, and the most important distinction in type is whether a calendar is single‐track or multi‐track. On a single‐track calendar, all students and teachers are on the same schedule (track). The school building either has all students present or none present on each day, and the building only has students in it 180 days per year. Single‐track YRE is usually implemented as an academic reform to improve student achievement. In contrast, multi‐track YRE is typically implemented in response to over‐crowding when there is no funding available for additional classroom space. On a multi‐track calendar, some of the students (for example, 25%) are on vacation at any time, while the other students (in this example, 75%) are in school. The tracks rotate through their time in school and on vacation, which would allow a school with room for 900 students to serve 1,200 students on a rotating basis.

Individual studies that examined both single‐ and multi‐track YRE have found that single‐track schools showed larger performance gains (e.g., White & Cantrell, 2001). The effect of multi‐track YRE may actually be negative (Graves, 2010; Graves, McMullen, & Rouse, 2013). In both the Kneese (1996) and Cooper et al. (2003) meta‐analyses, the authors found a larger treatment effect for single‐track than multi‐track YRE. Estimating the effect of grouped single‐ and multi‐track YRE as a single treatment of “year‐round education” would require ignoring the important guidance provided by prior research findings. As a result, the current study excludes multi‐track YRE and focuses only on single‐track YRE, because it is an academic intervention previously shown to have a modest but significant effect.

#### Types of outcome measures

The outcomes for this meta‐analysis will be (i) math achievement scores and (ii) reading achievement scores. If possible, supplementary analyses will examine growth instead of single‐year achievement scores, but initial review indicates that growth scores are not consistently available in studies that will be included in the final sample.

#### Types of settings

Studies cannot be evaluations of extended instructional time (e.g., lengthened school day or additional instructional days). It is not infrequent for schools or school districts to make multiple changes at once. However, it would not be possible to identify what share of a change in student performance was due to a year‐round calendar (i.e. the elimination of summer learning loss) and what share was due to additional days of instruction. I therefore plan only to include studies of schools on year‐round calendars but without extended instructional time.

### Search strategy

I will conduct searches using the following databases:

- ERIC
- PsycARTICLES
- PsycEXTRA
- PsychINFO
- ProQuest Research Library
- ProQuest Dissertations & Theses Global
- Dissertations & Theses @ CIC Institutions‎
- Education Administration Abstracts
- Education Full Text
- Social Sciences Citation Index
- Sociological Abstracts
- PolicyFile
- International Bibliography of the Social Sciences
- Periodicals Index Online
- EconLit
- Sociology Database
- PRISMA
- *Social Services Abstracts*
- *PAIS International*
- Google Scholar
- Google [for identifying grey literature; intending to review the first 400 results]
- Web of Science

My general/starting‐point search terms for this meta‐analysis include those used by Cooper et al. (2003), augmented by terms used in pertinent research published after that meta‐analysis. The basic form of the search terms is: “year‐round school*” or “year‐round education” or (school AND (“alternative calendar” or “modified calendar” or “balanced calendar”) or (“year‐round calendar” AND school). I will modify the precise terms, Boolean terms, etc. in order to take advantage of the search features, index terms identified in the resource's thesaurus, and tools within each of the above resources. Searches will be restricted to studies dated 2001–2016, in order to avoid duplicative inclusion of studies that were in the Cooper et al. (2003) work. In order to ensure that coding is being done well, a second researcher will code 10–25% of search results and 10–25% of full‐text downloads, to assess if reliability (both on studies to download and studies to include in final sample) is at least 90%. As searching is conducted, records will be saved in Mendeley for each search result, which will allow for clear indication of which results were found by each database/tool (for both sources found in multiple sources, and for unique results). Additionally, I will record the reason(s) that studies failed to meet study criteria.

In addition to searching databases, my research synthesis protocol includes footnote chasing in two directions. Using the “cited by” feature on both ProQuest and Google Scholar, I will examine all publicly available works that cited the Cooper et al. (2003) meta‐analysis or any study added to the final sample (sometimes called ‘cited reference searching’). Additionally, for each study that met the selection criteria, all footnotes will be reviewed and any studies that were not already part of the sample will be added from this traditional footnote chasing.

Finally, I will conduct searches or review the titles of all reports (depending on number of reports and available search interface on individual, e.g. corporate, websites) to identify additional grey literature at pertinent sites. Those sites will include the more than 50 (excluding higher education‐specific resources) listed in the Campbell information retrieval guide (Hammerstrøm, Wade, & Jørgensen 2010).

### Description of methods used in primary research

Expected methods include various combinations of the following:

- School‐level achievement, student‐level achievement
- School‐level matching to similar schools; student‐level matching (unlikely); comparison to geographically proximate schools; comparison to cohort preceding reform; exploitation of ‘natural experiment’ where external policy forces a change in a subset of similar schools; school‐within‐a‐school comparison
- Comparison of raw mean (and mean differences) of achievement scores for treated and comparison groups using t‐tests, ANOVA, ANCOVA, or MANOVA; comparison of percent proficient of treated and comparison groups; regression analysis (OLS).

### Criteria for determination of independent findings

For a meta‐analysis of single‐track YRE, the final sample is likely to be small, but to include studies from various states during the16 years examined. In this case it will be straightforward to assess whether the state‐by‐year population of any studies matches. In this unlikely case, I will contact authors to further assess whether their sample is the same, and only include a single instance of analysis for each school/year combination.

Several typical techniques for resolving within‐study dependence are not suitable to the single‐track YRE effect sizes. Multivariate meta‐analysis is the most common approach for addressing dependence among estimates (see Gleser & Olkin, 2009; Hedges & Olkin, 1985; Raudenbush, Becker, & Kalaian, 1988), but it requires within‐study correlation statistics (Becker, Hedges, & Pigott, 2004; Jackson, Riley, & White, 2011) which are not available for which are not available in any initially‐identified studies. Three‐level meta‐analysis may be able to account for hierarchically structured effect size estimates (Konstantopoulos, 2011), but there are unlikely to be sufficient estimates in this final sample for a three‐level model to be appropriate. Meta‐regression would also be mismatched without a larger sample of studies (Borenstein, Hedges, Higgins, & Rothstein, 2009).

The literature on YRE does not in general include effect sizes that are statistically dependent. For cases where I do encounter dependent effect sizes, I will conduct a sensitivity analyses where: (a) my main analyses exclude studies whose estimates are statistically dependent, (b) I conduct an alternative analysis where a single year of data for those studies is included in my estimate of an average effect size, and (c) an alternative analysis for if those studies' dependencies were ignored.

### Details of study coding categories

Since only one reviewer will be extracting data, the usual concern about reliability across coders does not apply. I will, for each study in the sample, extract the following fields (in addition to the data needed to calculate effect sizes for math and reading):

- Study Characteristics
  ○ Author
  ○ Year
  ○ Publication, dissertation/thesis, or report
  ○ State
  ○ Grade level(s)
  ○ Calendar structure
  ○ Summer break length
  ○ Racial data of students in sample
  ○ % eligible for free/reduced price lunch (or similar measure such as % poverty or % economically disadvantaged)
- Analysis Characteristics
  ○ Achievement test used
  ○ Psychometric category (standards based, norm‐referenced, criterion‐referenced)
  ○ Identification strategy employed
  ○ Analytic approach used

Some studies will likely meet all exclusion criteria and be part of my final sample, but not provide data that can be used to calculate an effect size. This most likely may include studies that omit key figures (e.g. a study that reports mean scores but not the associated standard deviations for the treatment and comparison groups). For such studies, I will report the direction and significance of their findings in a table dedicated to that purpose.

### Statistical procedures and conventions

I will extract the student outcome data needed for calculating the (Cohen's d) effect size(s) from each study. Cohen's d is the difference in outcome between the treatment and control groups divided by their pooled standard deviation (Borenstein, 2009). Preliminary searching reveals that in most cases this will be from mean score, standard deviation, and sample size (N) for the treatment and control groups. For other studies I will extract a reported effect size or calculate one from other reported data, such as F‐test results. In addition to full‐school statistics, where available, I will extract the data necessary for calculating effect sizes for sub‐groups of the full sample, for low‐SES students only and for minority students only. Preliminary searching indicates that most samples are over 100 and none are under 50, so the small‐sample correction (to use Hedges' g) is not likely to be needed (Hedges, 1981).

My main meta‐analytic calculation will be a weighted average (inverse variance) of a single estimate drawn from or calculated for each study in my final sample. Authors of meta‐analyses commonly calculate a simple or weighted average of multiple effects size estimates from a study in order to produce a single estimate for that study (used in 42.9% of meta‐analyses according to Ahn, Ames, & Myers, (2012); e.g. van Steensel, McElvany, Kurvurs, & Herppich, 2011). I plan to test for heterogeneity among the effect size estimates provided by the studies in my final sample, using Q‐statistics. However, even if (as current analysis indicates) the heterogeneity present in the estimates would normally point toward using random effects analysis, it is unlikely that the final sample will be large enough to meet the other assumptions of representativeness undergirding a random effects model. I plan to therefore provide both fixed effects (as more appropriate to the representativeness of the estimates) and random effects (as indicated by the Q‐statistics) estimates in my reporting of results.

Because of the observational nature of analyses of YRE, a formal bias categorization is likely inappropriate. I will assess publication bias. Many single‐track YRE evaluations are initially written as dissertation. I will take advantage of that – and the large array of descriptive and analysis tables typically included in dissertations – to always consult the dissertation as a primary document and published subsequent analyses of the same data as supplements, which should minimize the impact of analyses omitted from published works. I will also conduct at least one sensitivity analysis to inform my conclusions around bias based on study design; assessing whether there are differences in findings based on matching approach used (which appears to be the primary distinguishing point in study design in single‐track YRE).

### Treatment of qualitative research

I do not plan to include qualitative research.

## Review authors

**Lead review author:**

**Table:** 

Name: Dan Fitzpatrick
Title: Doctoral Candidate
Affiliation: Michigan State University
Address: 620 Farm Lane
City, State, Province or County: East Lansing, MI
Post code: 48825
Country: United States
Phone: 603‐438‐6026
Email: fitzpa88@gmail.com

Name: Jason Burns
Title: Doctoral Candidate
Affiliation: Michigan State University
Address: 620 Farm Lane
City, State, Province or County: East Lansing, MI
Post code: 48825
Country: United States
Phone: 330‐936‐9358
Email: burnsja6@msu.edu

## Roles and responsibilities

Because of the narrowly‐targeted scope of this review, the project is of a reasonable scope for a single author. Since I initially began reviewing single‐track year‐round education in 2012, I have developed content knowledge sufficient for completing the review. While he is not an author, for questions about systematic review methods, I have consulted Spyros Konstantopoulos as needed. The analyses of the effect of single‐track YRE rarely involve advanced statistical methods. If I do encounter a methodological practice or question that is beyond my own abilities, I will consult with Ken Frank.

## Sources of support

No support other than from Campbell.

## Declarations of interest

None

## Preliminary timeframe

30/9/17

## Plans for updating the review

I will remain responsible for updating this review every five years or will be available to help Campbell in identifying a new researcher to conduct the update.

## AUTHOR DECLARATION

### Authors' responsibilities

By completing this form, you accept responsibility for preparing, maintaining and updating the review in accordance with Campbell Collaboration policy. The Campbell Collaboration will provide as much support as possible to assist with the preparation of the review.

A draft review must be submitted to the relevant Coordinating Group within two years of protocol publication. If drafts are not submitted before the agreed deadlines, or if we are unable to contact you for an extended period, the relevant Coordinating Group has the right to de‐register the title or transfer the title to alternative authors. The Coordinating Group also has the right to de‐register or transfer the title if it does not meet the standards of the Coordinating Group and/or the Campbell Collaboration.

You accept responsibility for maintaining the review in light of new evidence, comments and criticisms, and other developments, and updating the review at least once every five years, or, if requested, transferring responsibility for maintaining the review to others as agreed with the Coordinating Group.

### Publication in the Campbell Library

The support of the Coordinating Group in preparing your review is conditional upon your agreement to publish the protocol, finished review, and subsequent updates in the Campbell Library. The Campbell Collaboration places no restrictions on publication of the findings of a Campbell systematic review in a more abbreviated form as a journal article either before or after the publication of the monograph version in Campbell Systematic Reviews. Some journals, however, have restrictions that preclude publication of findings that have been, or will be, reported elsewhere and authors considering publication in such a journal should be aware of possible conflict with publication of the monograph version in Campbell Systematic Reviews. Publication in a journal after publication or in press status in Campbell Systematic Reviews should acknowledge the Campbell version and include a citation to it. Note that systematic reviews published in Campbell Systematic Reviews and co‐registered with the Cochrane Collaboration may have additional requirements or restrictions for co‐publication. Review authors accept responsibility for meeting any co‐publication requirements.

**I understand the commitment required to undertake a Campbell review, and agree to publish in the Campbell Library. Signed on behalf of the authors**:

**Form completed by: Dan Fitzpatrick           Date: 16 May 2017**
